# Reduced lipolysis in lipoma phenocopies lipid accumulation in obesity

**DOI:** 10.1038/s41366-020-00716-y

**Published:** 2020-11-24

**Authors:** Diana Le Duc, Chen-Ching Lin, Yulia Popkova, Zuqin Yang, Velluva Akhil, M. Volkan Çakir, Sonja Grunewald, Jan-Christoph Simon, Andreas Dietz, Dirk Dannenberger, Antje Garten, Johannes R. Lemke, Jürgen Schiller, Matthias Blüher, Pamela Arielle Nono Nankam, Ulrike Rolle-Kampczyk, Martin von Bergen, Janet Kelso, Torsten Schöneberg

**Affiliations:** 1 Institute of Human Genetics, University Medical Center Leipzig, 04103 Leipzig, Germany; 2 Department of Evolutionary Genetics, Max Planck Institute for Evolutionary Anthropology, 04103 Leipzig, Germany; 3 Institute of Biomedical Informatics, National Yang-Ming University, Taipei, 11221 Taiwan; 4 Institute for Medical Physics and Biophysics, Medical Faculty, Leipzig University, 04107 Leipzig, Germany; 5 Division of Molecular Biochemistry, Rudolf Schönheimer Institute of Biochemistry, Medical Faculty, Leipzig University, 04103 Leipzig, Germany; 6 Department of Dermatology, Venereology and Allergology, University Medical Center Leipzig, 04103 Leipzig, Germany; 7 Clinic for Otorhinolaryngology, Head and Neck Surgery, University Medical Center Leipzig, 04103 Leipzig, Germany; 8 Leibniz Institute for Farm Animal Biology, Institute of Muscle Biology and Growth, Wilhelm-Stahl-Allee 2, 18196 Dummerstorf, Germany; 9 Pediatric Research Center, University Hospital for Children and Adolescents, Leipzig University, 04103 Leipzig, Germany; 10 Helmholtz Institute for Metabolic Obesity and Vascular Research (HI-MAG) of the Helmholtz Zentrum München at the University of Leipzig and University Hospital Leipzig, Leipzig, Germany; 11 Department of Endocrinology, Faculty of Medicine, University of Leipzig, 04103 Leipzig, Germany; 12 Department of Molecular Systems Biology, Helmholtz Centre for Environmental Research GmbH, 04318 Leipzig, Germany; 13 Institute of Biochemistry, Faculty of Life Sciences, Leipzig University, Bruederstr. 32, 04103 Leipzig, Germany

**Keywords:** Translational research, Preclinical research

## Abstract

**Background:**

Elucidation of lipid metabolism and accumulation mechanisms is of paramount importance to understanding obesity and unveiling therapeutic targets. In vitro cell models have been extensively used for these purposes, yet, they do not entirely reflect the in vivo setup. Conventional lipomas, characterized by the presence of mature adipocytes and increased adipogenesis, could overcome the drawbacks of cell cultures. Also, they have the unique advantage of easily accessible matched controls in the form of subcutaneous adipose tissue (SAT) from the same individual. We aimed to determine whether lipomas are a good model to understand lipid accumulation.

**Methods:**

We histologically compared lipomas and control SAT, followed by assessment of the lipidome using high-resolution ^1^H NMR spectroscopy and ESI-IT mass spectrometry. RNA-sequencing was used to obtain the transcriptome of lipomas and the matched SAT.

**Results:**

We found a significant increase of small-size (maximal axis < 70 µm) and very big (maximal axis > 150 µm) adipocytes within lipomas. This suggests both enhanced adipocyte proliferation and increased lipid accumulation. We further show that there is no significant change in the lipid composition compared to matched SAT. To better delineate the pathophysiology of lipid accumulation, we considered two groups with different genetic backgrounds: (1) lipomas with *HMGA2* fusions and (2) without gene fusions. To reduce the search space for genes that are relevant for lipid pathophysiology, we focused on the overlapping differentially expressed (DE) genes between the two groups. Gene Ontology analysis revealed that DE genes are enriched in pathways related to lipid accumulation.

**Conclusions:**

We show that the common shared lipid accumulation mechanism in lipoma is a reduction in lipolysis, with most gene dysregulations leading to a reduced cAMP in the adipocyte. Superficial lipomas could thus be used as a model for lipid accumulation through altered lipolysis as found in obese patients.

## Introduction

Obesity is one of the most significant health burdens worldwide. It occurs through an excessive accumulation of fat in adipocytes via increased cell volume (hypertrophy), cell number (hyperplasia), or a combination of both [1]. Animal models have been intensively used to improve our understanding of the pathology [2], however, in vitro studies are of paramount importance for the dissection of the underlying molecular mechanisms [1]. Moreover, an in vitro setup allows using human material, which facilitates the applicability of the results toward the human disease. While these models enable a controlled investigation of adipogenesis regulators, they do not entirely reflect in vivo adipogenesis, since the in vitro setup requires a series of defined adipogenic cocktails for differentiation or fat accumulation [3]. E.g., a PPARγ agonist together with the isobutylmethylxanthine/dexamethasone/insulin differentiation cocktail increases leptin mRNA levels [4], which may not necessarily reflect the in vivo status. Thus, a better representation of in vivo mechanisms is offered by direct inquiry of adipose tissue from obese patients compared to controls. The major drawback here is that it is impossible to control for donor-related factors or environmental exposure, which would facilitate a better understanding of adipogenesis and lipid accumulation triggers.

Lipoma could be a human model that allows the investigation of lipid accumulation mechanisms, reflects in vivo processes with high fidelity, and permits to control for donor-related and environmental factors. Hence, we inquired lipid accumulation mechanisms in different subtypes of conventional lipomas together with subcutaneous adipose tissue (SAT) collected from the same individual.

Phenotypically, conventional lipomas are very homogenous and often present very large adipocytes (up to 300 µm) containing one lipid droplet [5]. Genetically they are, however, quite heterogeneous [6, 7]. The most common chromosomal rearrangement in lipomas involves fusions of *HMGA2*; while, chimeric genes derived from the fusion of *HMGA2* with multiple different partners were suggested to play a role in lipomatous development [8, 9], the same gene fusions have been identified in other benign mesenchymal tumors, such as chondromas, with no lipid accumulation [10–12]. This suggests that fusion genes may be implicated in tumor promotion, but are not necessarily related to lipid accumulation.

Given the common phenotype, we hypothesize that distinct genetic subtypes will have a common mechanism of lipid accumulation. Based on gene expression profiles, we show that the overlap of differentially expressed (DE) genes between lipomas with *HMGA2* fusions and without any fusion genes is significant, supporting our initial hypothesis. To reduce the search space, we further focused on lipid composition and pathways shared between the two subgroups, which are relevant for lipid storage. We finally demonstrate that lipoma is a valid model to understand and potentially modulate lipid accumulation.

## Materials and methods

### Patients and samples collection

We recruited 15 patients (12 males and 3 females; age range 26–79 years; normal BMI) with single superficial subcutaneous lipomas, located as follows: four on the abdomen, four on the trunk, six on the arm, and one on the upper thigh. Lipomas were surgically removed in the Clinics for Dermatology and for Otorhinolaryngology at the University Hospital Leipzig. During the surgical procedure, normal adipose tissue was excised from the adjacent region to be further analyzed as a matched control. To preserve the aesthetics of the region, we could only collect a limited amount of control tissue. SAT is difficult to discriminate from dermal adipose tissue in humans. However, a principal component analysis of expression profiles from the control samples did not reveal a specific clustering based on the region where normal adipose tissue was collected. Therefore, we considered these samples collected from the abdomen as SAT, and since the other samples showed no specific clustering, all controls were denoted to be SAT.

All procedures in this study were approved and monitored by the ethic committee of Leipzig University, Germany (380/16-ek). Informed consent was obtained from all subjects. Tissue sections of each sample were examined by light microscopy after hematoxylin and eosin staining to confirm that lipomas were the conventional type without any cellular or nuclear atypia.

Depending on the quantity of the normal SAT that could be collected we prioritized RNA sequencing (RNA-seq) experiments rather than the analysis of the lipid and metabolic compounds. The rationale is that RNA-seq offers a broader molecular characterization of the samples. Thus, 10 matched samples underwent both lipid composition and RNA-seq analyses, while RNA-seq alone was performed for 15 matched samples.

### Histologic evaluation

The histologic evaluation could not be performed on matched lipomas and SAT, since the amount of normal SAT collected had to be minimal, in order to preserve the aesthetics of the region. As control, we selected ten representative donors (normal BMI; gender: five women and five men; mean age: 51.5 ± 17.3 years) from our previously reported human adipose tissue biobank [13]. Harvested adipose tissues were formalin-fixed and paraffin-embedded. We prepared four to five 3.5 µm-thick sections per adipose tissue donor.

Adipocyte size distribution was analyzed using a Keyence BZ-X800 microscope and BZ-X800 Analyzer software (Keyence Corp., Osaka, Japan) following the manufacturer instructions. We considered only samples that showed no alterations in adipocyte structure like leakage of the lipid droplet or membrane ruptures. Approximately 6000–9000 adipocytes were analyzed per adipose tissue donor from the control (*n* = 10) and lipoma group (*n* = 11).

Statistical testing on differences in the average of adipocytes population was performed using a Welch *t* test in R. Differences in the distribution of adipocyte size were tested using the Kolmogorov–Smirnov test in R.

### Lipid and metabolic compounds

For a qualitative investigation of the fat tissue, we subjected *n* = 10 untreated lipoma samples and the matched normal SAT to the procedures described in the following sections. A detailed description of the lipid analysis methods is available in the Supplementary Material.

#### Lipid extraction

Tissue samples were extracted using methyl-*tert*-butyl ether following the protocol of Matyash et al. [14] with slight modifications.

#### High-performance thin-layer chromatography (HPTLC) and electrospray ionization ion trap mass spectrometry (ESI-IT MS)

To overcome potential suppression effects, crude lipid extracts were separated by HPTLC and the individual lipid fractions triacylglycerol (TAG), phosphatidylcholine (PC), and sphingomyelin (SM) were subsequently analyzed by means of ESI-IT MS. HPTLC and ESI-IT MS measurements were performed as previously described [15].

#### Proton nuclear magnetic resonance (^1^H NMR)

^1^H NMR measurements were performed on a Bruker AVANCE-700 (Bruker, Rheinstetten, Germany) spectrometer operating at 700.13 MHz for ^1^H. All spectra were recorded at 310 K using a 5-mm inverse probe and the sample volume was 450 μl in all cases. All spectra were corrected for baseline and phase distortions and calibrated using the residual proton resonance of methanol at 3.49 ppm. The lipid composition of the samples was determined by integrating the methyl (0.9 ppm), allylic (2.7 ppm), olefinic (5.3 ppm), and vicinal-olefinic (2.0 ppm) resonances.

#### Gas chromatography flame-ionization detection (GC-FID)

A detailed fatty acid analysis was performed as previously described [16] using capillary GC with a CP-Sil 88 CB column (100 m × 0.25 mm, Chrompack-Varian, Lake Forest, CA, USA) that was installed in a Perkin Elmer gas chromatograph Autosys XL with a flame-ionization detector and split injection (Perkin Elmer Instruments, Shelton, USA).

### RNA extraction and sequencing

For library preparation and sequencing, total RNA was extracted from *n* = 15 lipomas and the matched normal SAT using RNeasy® Lipid Tissue Kit (Qiagen). Indexed cDNA libraries were constructed using the TruSeq RNA sample preparation kits v2 (Illumina, San Diego, CA). Libraries were sequenced on an Illumina HiSeq platform as 101 bp paired-end reads to an average of 46.9 million reads per library.

### Processing of RNA reads

RNA-seq reads were demultiplexed, trimmed of adapters, and mapped with STAR (version 2.6.1d) [17] to the GRCh38 genome assembly. We used additional options to default parameters (Supplementary Material) to detect gene fusions and increase the sensitivity for novel splice junctions.

#### Gene fusion analysis

Arriba (https://github.com/suhrig/arriba/) was used to detect gene fusions from RNA-seq data. Briefly, the fusion candidates are generated by STAR [17] and collected in the chimeric alignment files. Arriba was run sequentially on all samples and only fusion events with high confidence level were considered (Supplementary Material).

#### Differential gene expression analysis

The transcription level of each gene was determined using htseq-count [18] followed by differential analysis on gene count data with DESeq2 [19].

Gene expression analysis was run on the two groups, lipoma and SAT, accounting for the matched samples. The initial analysis was performed using all *n* = 15 samples to test whether the inclusion of potential multiple genomic backgrounds, and potential different pathomechanisms, may lead to additional noise. We further focused the analysis on two lipoma groups: with (*n* = 4) and without (*n*  =  4) *HMGA2* fusion genes. We considered shared up- and downregulated genes between lipomas without fusions and with *HMGA2* fusions and corrected for multiple testing using the Benjamini–Hochberg method.

#### Protein–protein interaction (PPI) networks and Gene Ontology (GO) analysis

To better understand the pathomechanism of lipoma, conventional functional enrichment analysis for DE genes was performed. Functional annotations of genes were obtained from GO [20, 21].

A protein interaction and a network-wise functional enrichment analysis were incorporated to discover functional modules within DE genes (*p*-value < 0.05) [22] (Supplementary Material). These two *p*-values were adjusted by the Benjamini–Hochberg multiple testing procedures to control the false discovery rate (FDR) [23].

We aimed to identify shared functional modules between lipomas without fusions and with *HMGA2* fusions. To increase the power of functional module identification, the protein interaction partners of the common DE genes were incorporated.

To further determine which pathways are significantly dysregulated, an enrichment analysis of GO biological process categories was performed [24]. We considered the results to be significant for an FDR < 0.05.

We finally identified genes in the KEGG [25] pathway regulation of lipolysis in adipocytes (map 04923) that showed DE in lipomas without fusions and the *HMGA2*-fusion group. The expression profile of the gene classes (e.g., genes coding for phosphodiesterases) was considered to follow a binomial distribution from which we derived the probability of observing DE genes (Supplementary Material).

## Results

### Adipocyte size evaluation

**Fig. 1:**
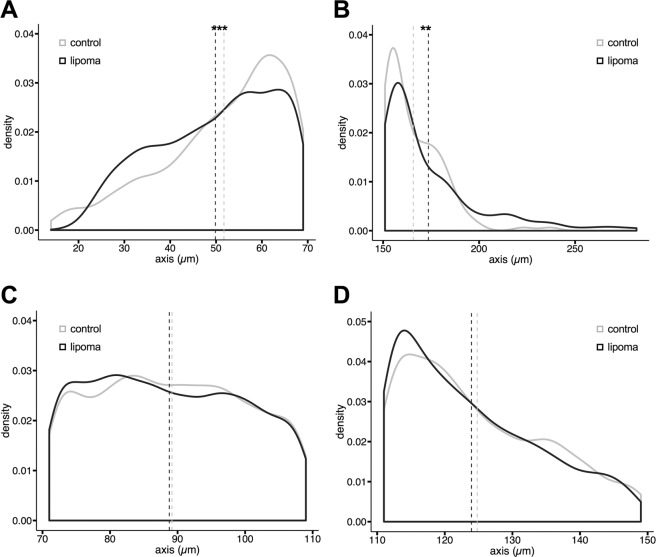
Density distributions of adipocyte populations. **A** There is a significant increase of small-size (major axis < 70 µm) and **B** very large adipocytes (major axis > 150 µm) in lipomas. **C** Normal (major axis 70–110 µm) to **D** large (major axis 110–150 µm) adipocytes show no significant difference in lipomas *vs*. normal SAT. ***p-value < 0.001; **p-value < 0.01. *n* lipoma = 11, *n* SAT = 10, 6000–9000 adipocytes were analyzed from four to five histological sections per sample.

A previous small study (*n* = 5) that looked at lipoma histology compared to normal SAT revealed an increase of small adipocytes, which were related to increased adipogenesis in lipomas [26]. To test this in our samples, we used fixed tissue sections from 15 lipoma patients and compared them to SAT from ten representative donors from our previously reported human adipose tissue biobank [13]. Our results confirm the study of Suga et al. [26] and show a significant difference for small-size adipocyte population (maximal axis < 70 µm) both in the density distribution (Fig. 1A, *p*-value = 5.05e−08) and in the average maximal axis value (*p*-value = 5.639e−07; lipoma = 49.85 ± 0.22 µm; SAT = 51.72 ± 0.31 µm). There is no significant difference in the normal size adipocytes (maximal axis 70–110 µm, Fig. 1C), while larger adipocytes (110–150 µm) show marginally significant difference in the density distribution (Fig. 1D, *p*-value  = 0.052) and average maximal axis value (*p*-value = 0.043; lipoma = 123.94 ± 0.28 µm; SAT = 124.79 ± 0.32 µm). Yet, we further observed a significant difference in the very large adipocyte population with a higher lipoma adipocyte size (Fig. 1B, *p*-value = 0.002; lipoma = 173.59 ± 1.31 µm; SAT = 165.70 ± 2.13 µm). Therefore, in accordance with Suga et al. [26] lipomas display smaller size adipocytes, which suggests enhanced adipogenesis, but also an increase in very large adipocytes, most probably as a result of increased lipid accumulation.

Additionally, concomitantly to the findings of Suga et al. [26], we also did not observe differences in inflammatory infiltrates or fibroblasts between lipoma and control SAT.

### Lipid and metabolic compounds

**Fig. 2:**
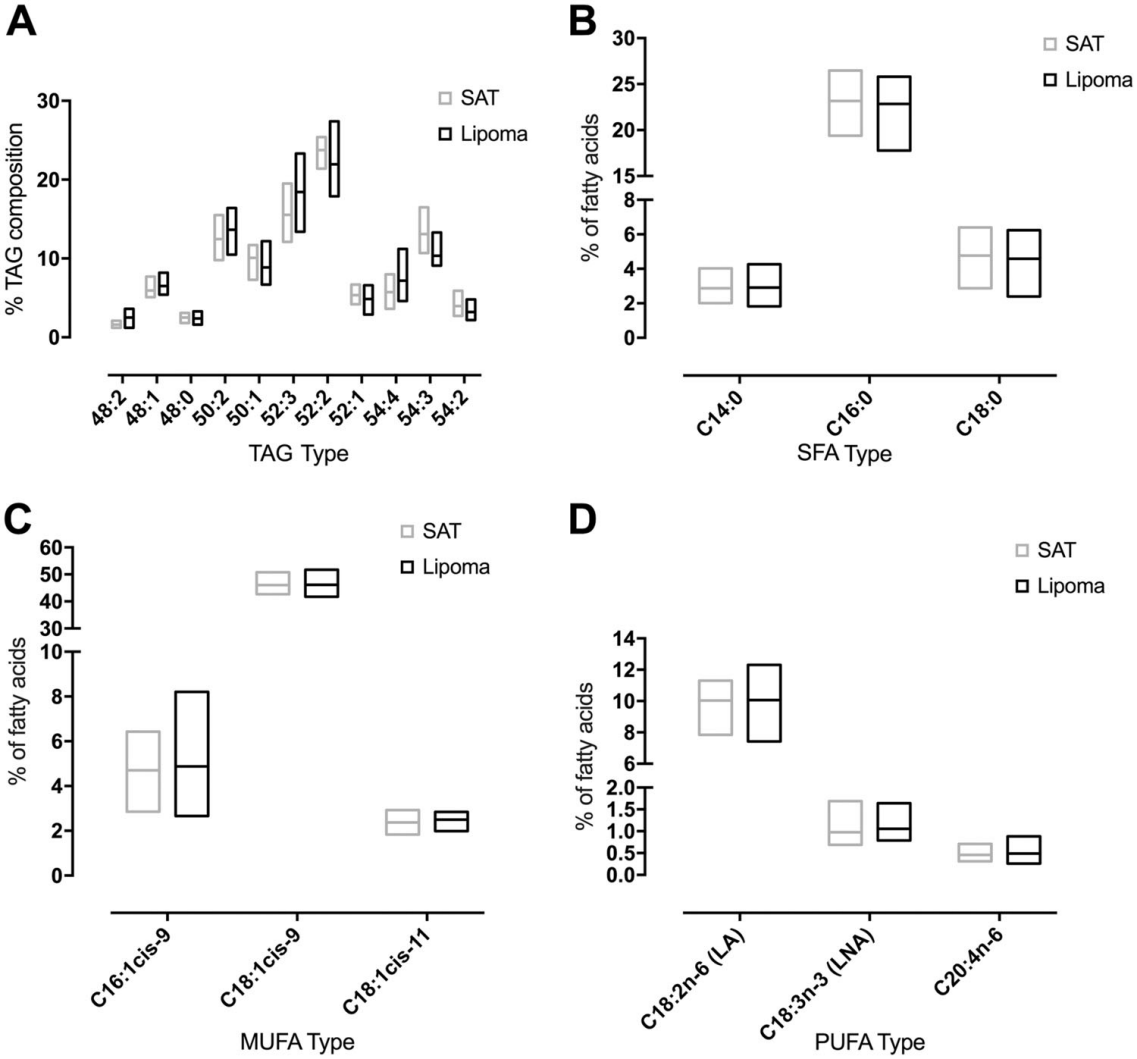
Lipidomics analysis of lipoma *vs*. matched SAT. **A** Relative intensity of TAGs in lipoma and SAT. The relative intensities in percent were generated from ESI-IT MS data (*n* = 10); the bars extend from minimum to maximum and the horizontal line depicts the mean. **B**–**D** GC-FID determination of the relative fatty acid composition of SAT and matched lipoma extracts (*n* = 10). For the most abundant **B** saturated fatty acids (SFA), **C** monounsaturated fatty acids (MUFA), **D** polyunsaturated fatty acids (PUFA) the tissue type could not explain the difference among samples (two-way ANOVA; *p*-value = 0.35; *p*-value = 0.55; *p*-value = 0.41, respectively).

To check whether the different adipocyte populations result in different lipid composition, we analyzed lipoma *vs*. matched normal SAT by different lipidomics techniques. Major lipids detected were TAGs. Relative intensities of selected TAG species are shown in Fig. 2A. There is a trend toward longer-chained TAGs with higher double bond content. Representative ESI-IT MS spectra of organic extracts of SAT and corresponding lipoma tissue are shown in the Supplementary Fig. S1 and assignments of all detected *m/z* ratios are summarized in Supplementary Material (Supplementary Table S1).

To obtain detailed information about fatty acid compositions of the assigned TAGs, we performed GC analysis. Most abundant saturated, monounsaturated, and polyunsaturated fatty acids are summed up in Fig. 1B–D. As in case of ESI-IT MS, only not significant differences in the distribution of fatty acyl residues were observed. Most abundant fatty acids were palmitic acid (C16:0), oleic acid (C18:1cis-9), and linoleic acid (C18:2n-6).

To investigate possible quantitative changes regarding TAG content, we analyzed organic extracts of SAT and lipoma via ^1^H NMR. The spectra are dominated by TAGs and resonances of most relevant functional groups (content of double bonds and allylic protons) can be easily assigned. As in the case of ESI-IT MS, ^1^H NMR spectra also show no significant differences between lipoma and matched SAT samples (Supplementary Material, Supplementary Fig. S2).

An analysis of 145 metabolites from three compound classes (acyl carnitines, glycerophospholipids, and sphingolipids) together with ESI-IT MS of membrane lipids (PCs and SMs) showed no significant differences between lipomas and matched SAT (Supplementary Material, Supplementary Table S2 and Figs. S3 and S4).

### Gene fusion analysis

**Fig. 3:**
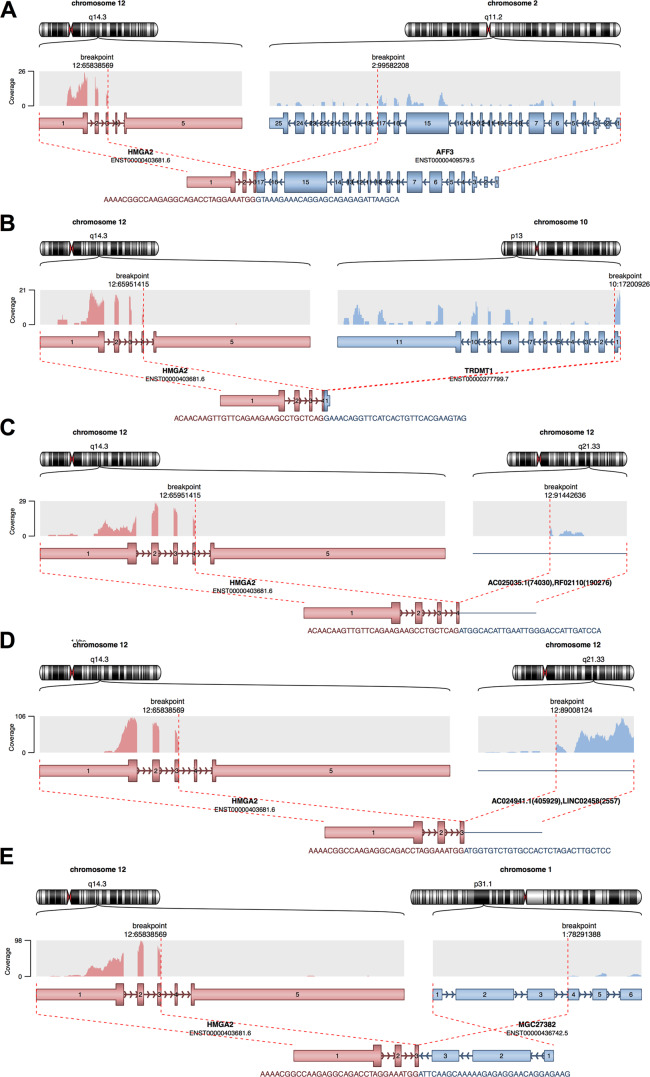
High-confidence fusion events. Fusions detected in lipomas (**A**–**D**) and in one SAT sample (**E**), which is matched to the lipoma sample (**B**). Fusions between *HMGA2* and other partner genes involve two different chromosomes (**A**, **B**, **E**) or only chromosome 12 (**C**, **D**). The fusion breakpoints and the resulting product are shown in red (*HMGA2*) and blue (fusion partner).

Since gene fusions have been suggested to play a major role in lipoma etiology [8, 9], we explored gene fusion events supported by RNA-seq data. To this end, we identified reads spanning two genes or reads located in the fusion junction for each sample separately. Considering only high-confidence events (defined in Supplementary Material), only 1 of the 15 SAT samples displayed a gene fusion event, compared to 7 of the lipoma samples (Supplementary Table S3, Gene fusions). Of the seven lipomas with high-confidence events, four displayed *HMGA2* fusions (Fig. 3). From the rest of lipoma samples, four displayed no fusions, while other four showed medium confidence fusion events, none of which involved *HMGA2* gene. Fusion partners of *HMGA2* included genes located on different chromosomes (Fig. 3). Interestingly, the only high-confidence fusion identified in the matched SAT samples also involved *HMGA2* and a partner located on chromosome 1 (Fig. 3E). This sample comes from the same patient as the lipoma sample depicted in Fig. 3B, which suggests instability of the *HMGA2* complex, since in the normal tissue there appears a different fusion event.

An analysis of differential splicing events showed no significant differences between lipoma and SAT (Supplementary Fig. S5).

### Differential gene expression analysis

We hypothesized that the different genetic backgrounds play a role in tumorigenic transformation. Thus, we considered that lipomas with *HMGA2* fusions and the ones without fusions arise through a different mechanism, but have a common dysregulation of fat accumulation, and hence the similar phenotype. If samples spanned different genetic backgrounds, the analysis would result in multiple DE genes, with increased noise, since there are different triggers for adipocyte proliferation (tumorigenic transformation).

**Fig. 4:**
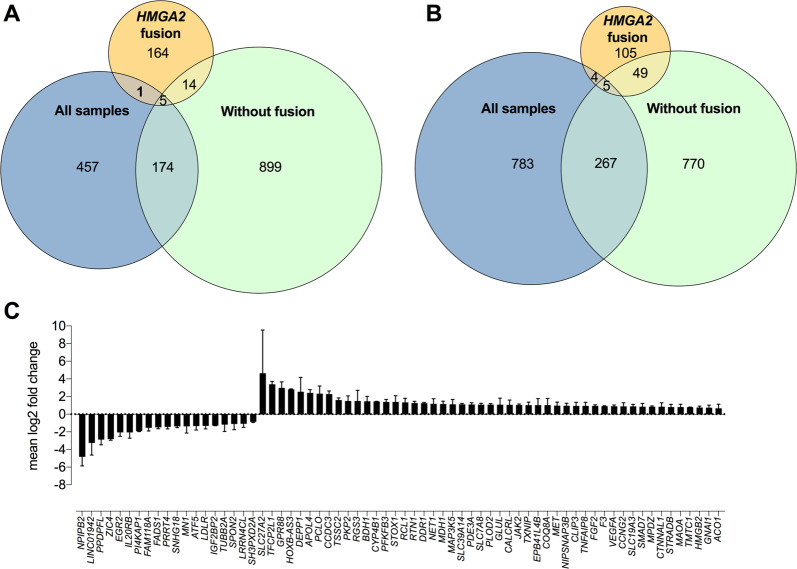
DE genes. Analyses including all samples (*n* = 15), lipomas without gene fusions (*n* = 4), and lipomas with *HMGA2* fusion (*n* = 4). **A** Venn diagram of downregulated genes. **B** Venn diagram of upregulated genes. **C** Shared DE genes between lipomas with *HGMA2*-fusion and lipomas without fusions. *p* adj was corrected for multiple testing using Benjamini–Hochberg method and considering all genes expressed in adipose tissue.

To raise evidence for our hypothesis, we initially tested differential gene expression on all 15 lipoma samples and compared the results to the group with *HMGA2* fusions, which had a similar genetic background. Indeed, we observed 637 significantly downregulated and 1059 upregulated genes in lipoma *vs*. matched normal SAT (Figs. 4A, B and Supplementary Table S3, DEG all samples) when we tested all samples together. Conversely, testing the *HMGA2* fusions group with a narrowed genetic background resulted in far less DE genes, with a total of 342, of which 184 were downregulated and 158 were upregulated (Figs. 4A, B and Supplementary Table S3, DEG *HMGA2* fusion).

Since this matched our initial hypothesis, we decided to focus our analysis on the two groups: lipomas without any fusions (Supplementary Table S3, DEG no-fusion) and with *HMGA2* fusion. Because the resulting phenotype is the same, namely, fat accumulation, we considered the overlapping genes between the two groups to be causative for this convergence. This resulted in 19 downregulated and 54 upregulated genes (Fig. 4C). This overrepresentation of shared DE genes (*p*-value < 0.01, Fisher’s exact test) suggests a common mechanism between the two types of lipomas. Additionally, we observed significantly more shared DE genes between no-fusion and all lipomas (*p*-value < 0.01, Fisher’s exact test), while the proportion of intersection with *HMGA2* fusion was small and did not reach significance (*p*-value > 0.4, Fisher’s exact test, Figs. 4A, B). This implies that the majority of the lipomas in our samples act like no-fusion lipomas, while *HMGA2*-fusion lipomas can be considered a specific subtype.

We additionally tested whether assigning the samples to the two different groups according to the gene fusion status has an impact on the lipid composition analysis. This analysis also showed no significant qualitative changes.

An analysis of transcription factors that could be responsible for the expression profile showed no significant differences between lipoma and SAT (Supplementary Fig. S6).

### Protein–protein interaction (PPI) networks and Gene Ontology analysis

We integrated PPIs of shared DE genes between lipomas with *HMGA2* fusions and the ones without fusions to identify shared dysregulated functional modules. Since the number of common DE genes is too small to run the functional enrichment analysis directly, we incorporated the protein interaction partners of the common DE genes to increase the power of functional modules identification (Supplementary Table S3, Common down PPI, Common up PPI). Lipid-related functional modules that are enriched among downregulated and upregulated genes, respectively, are presented in Table 1. Common downregulated genes appear to be involved in lipid metabolic processes and homeostasis, while upregulated genes are related to the negative regulation of lipid localization and storage.

**Table 1:** Enriched lipid-related functional modules in down- and upregulated genes.

GOID	Description	#Nodes	#Edges	Adj_N *p*-value	DE *p*-value
Downregulated					
GO:0019216	Regulation of lipid metabolic process	40	45	1.77e−05	1.59e−02
GO:0055088	Lipid homeostasis	12	8	1.46e−02	2.21e−02
Upregulated					
GO:1905953	Negative regulation of lipid localization	13	6	5.24e−03	1.45e−02
GO:0010888	Negative regulation of lipid storage	8	3	2.61e−03	4.01e−02

#Nodes: number of downregulated genes involved in the function; #Edges: number of PPI among downregulated/upregulated genes involved in the function; Adj_N *p*-value: adjusted *p*-value of the gene enrichment (Benjamini–Hochberg correction); DE *p*-value: *p*-value of the differentially downregulated/upregulated gene enrichment.

These results are further supported by the significant enrichment of GO categories like lipid metabolic process (GO:0006629, FDR = 0.007) or regulation of lipid localization (GO:1905952, FDR = 0.02). Additionally, DE genes were enriched in pathways related to adipose tissue development (GO:0060612, FDR = 0.005) and differentiation (GO:0045444, FDR = 0.01) (Supplementary Table S3, GO enrichment).

**Fig. 5:**
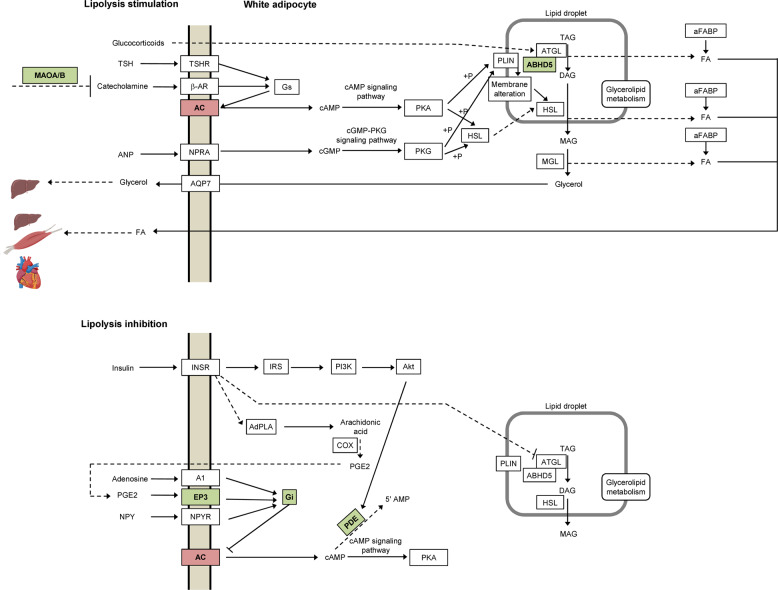
Regulation of lipolysis in adipocytes (after KEGG [25] map 04923). Lipolysis is stimulated by receptors coupled to stimulative regulative G protein (Gs, upper panel) and inhibited by receptors coupled to inhibitory regulative G protein (Gi, lower panel), which increase or decrease intracellular cAMP level, respectively. Of 27 gene clusters depicted in the pathway, 6 show differential gene expression (red downregulation, green upregulation in lipoma *vs*. SAT (subcutaneous adipose tissue)). The probability that this is a random event is 0.002. Genes that show differential expression influence the cAMP-signaling pathway and lead to a reduction in cAMP levels in the adipocyte.

Based on the shared DE genes, we observed a possible alteration of the lipolysis pathway. We thus inquired the KEGG annotation [25] of lipolysis in adipocytes. The probability that 6 of the 27 gene classes depicted in the pathway show DE (*p*-value < 0.05) by chance is 0.002. Additionally, we noticed that DE genes mainly influence the cAMP signaling pathway, which could lead to a cAMP reduction in the adipocyte (Fig. 5 and Supplementary Table S3, GPCRs coupling).

## Discussion

There are multiple cellular models, which have proven useful for evaluating adipogenesis and lipid accumulation. The 3T3-L1 murine cell line is still most used, because differentiation protocols are well-developed and standardized [1]. However, human cell lines, like adipose-derived stem cells (ASCs), have proven more useful for applications on human health because they overcome the physiological and metabolic differences between species [1]. Using ASCs to understand in vivo processes is still complicated by differentiation protocols or donor-related differences.

We, thus, sought to understand lipid accumulation processes using lipoma as model. This model better reflects the in vivo setup, since the tissue is not cultured or differentiated; at the same time lipoma permits to control for genetic and environmental confounding factors, because matched SAT from the same individual is easily accessible.

A previous study showed that in comparison to normal adipose tissue, lipomas have a bimodal distribution of adipocytes sizes, showing an increase in small-size adipocytes [26]. This was related to the presence of adipose-derived progenitor cells and enhanced adipogenesis [26]. However, lipomas are known to demonstrate variability in cell size, often with very large adipocytes (up to 300 µm) containing one lipid droplet [5]. Our results show significant differences in both small-size (<70 µm) and very large (>150 µm) adipocyte population distributions (Figs. 1A, B). This suggests that both adipogenesis and lipid accumulation may be disturbed in lipomas. Obese patients have been shown to have a significant increase in very large adipocytes, but with a concomitant decrease in the small adipocytes population [27]. Adipocyte hypertrophy is considered a major determinant of insulin resistance associated to obesity [27, 28].

To check whether the difference in adipocyte populations results in a change in lipid composition, we analyzed lipoma *vs*. matched normal SAT by different lipidomics techniques. This revealed no significant difference between lipomas and matched normal SAT (Fig. 2). Additionally, we also analyzed the composition of 145 metabolites and observed no significant differences as well (Supplementary Material, Supplementary Figs. S3 and S4).

Interestingly, while lipomas are phenotypically homogeneous, genetically they are very heterogenous and about two-thirds have chromosomal aberrations [29]. The most common rearrangements involve the *HMGA2* gene. Yet, there are multiple benign tumors like chondroid hamartomas of the lung [30–33], uterine leiomyomas or adenomas of the salivary glands [33, 34] that display *HMGA2* rearrangements, but do not accumulate fat. Thus, although *HMGA2* rearrangements appear with a high frequency in lipomas and have been implicated in lipomatous formation [8, 9], the rearrangement is more likely to play a role in tumor transformation and not in building the fat depots. We hypothesized that the genetic background plays a role in adipocyte proliferation, but not in the lipid accumulation. Yet, regardless of the proliferative trigger, all lipomas show fat accumulation, probably as a result of the same dysregulation.

To test our hypothesis, we inquired the gene expression profiles of 15 lipomas and their matched normal SAT. When we tested samples with multiple genetic backgrounds there were significantly more DE genes (Figs. 4A, B and Supplementary Table S3, DEG all samples) than when we tested only *HMGA2*-fusion lipomas (Figs. 4A, B and Supplementary Table S3, DEG *HMGA2* fusion). Based on the DE overlap we concluded that *HMGA2*-fusion lipomas are a specific molecular subtype, most probably because the adipocyte proliferation occurs through the same trigger in this subgroup.

Furthermore, the proportion of shared DE genes among lipomas with and without *HMGA2* fusion is significantly overrepresented (*p*-value < 0.01, Fisher’s exact test), supporting a common mechanism that may lead to the same phenotype. This enforces our initial assumption that while the tumor transformation may arise through different pathways, lipomas share a common lipid accumulation mechanism.

To understand how lipid accumulation occurs, we initially integrated PPIs with gene expression profiles and biological function annotations. This showed that downregulated genes cluster in lipid metabolic processes, while upregulated genes are involved in lipid localization and lipid storage (Table 1). While there are previous studies that analyzed expression profiles in lipomas in general [35, 36] or special subtypes [6, 37], none investigated the overlap between the different subtypes. A focus on the overlap allows a reduction in the search space to reveal relevant pathways dysregulated in lipomas with *HMGA2* and without any fusions.

In general, upregulated genes seem to be involved in lipid storage (Table 1). An upregulated gene, *MAOA*, encodes for the monoamine oxidase A, which catalyzes the oxidative deamination of biogenic amines including norepinephrine and epinephrine, the neurotransmitter that regulates sympathetic nervous system tone and adrenergic signaling. Norepinephrine and epinephrine control lipolysis through stimulation of adipocyte β adrenergic receptors, which leads to the generation of the second messenger cAMP in the cytosol and activates protein kinase A. In turn, lipolytic enzymes like hormone-sensitive lipase and adipose TAG lipase, which hydrolyze TAGs into glycerol and free fatty acids, are activated and recruited to the lipid droplet to induce lipolysis (Fig. 5). Higher MAOA levels lead to degradation of norepinephrine and epinephrine and reduced lipolysis. *MAOA* expression increases during 3T3-L1 cell differentiation [38] and white adipose tissue of obese dogs fed a high-fat diet [39]. Additionally, MAOA levels are predictive of body mass index changes in adolescents and young adults [40]. Similarly, *PDE3A*, a major cAMP degradation enzyme, is upregulated in lipoma acting synergistically together with the increase *MAOA* reducing the lipolytic tone in lipoma. Consistently, reduced *PDE3A* expression in omental adipose is associated with excess weight loss in patients with Roux-en-Y gastric bypass [41]. At a closer look of KEGG lipolysis pathway (Fig. 5), we observed a significant enrichment of differentially regulated genes mostly involved in the cAMP-signaling pathway, which leads to a reduction of cAMP levels in the adipocyte (Supplementary Table S3, GPCRs coupling). The main mechanism of fat accumulation appears to be a reduction in lipolysis, which has been previously reported to play a role in body fat accumulation in, e.g., the elderly, also through *MAOA* upregulation [42].

## Conclusion

In sum, we show based on gene expression profiles that *HMGA2*-fusion lipomas are a specific subtype and while, the fusion may promote tumorigenic transformation, all lipomas share a common fat accumulation mechanism. Our results point to lipid accumulation through altered lipolysis that does not result in major changes in lipid composition. This observation may aid using superficial lipomas as a model for lipid accumulation through altered lipolysis.

## Supplementary information

Supplemental Material

Supplementary Table S3

## Data Availability

RNA-Seq reads and expression profiles are deposited in Gene Expression Omnibus (http://www.ncbi.nlm.nih.gov/geo/) under project ID GSE141027.
