# Supplementary material for: Reduced lipolysis in lipoma phenocopies lipid accumulation in obesity
Source: Int J Obes (Lond). 2020 Nov 24;45(3):565–76. doi: 10.1038/s41366-020-00716-y (PMC7906903; doi:10.1038/s41366-020-00716-y)
Supplement: Supplementary file 1 — Supplemental Material [file 41366_2020_716_MOESM1_ESM.docx]

**Reduced lipolysis in lipoma phenocopies lipid accumulation in obesity**

Diana Le Duc ^1, 2, *^, Chen-Ching Lin ^3^, Yulia Popkova ^4^, Zuqin Yang ^5^, Velluva Akhil ^2, 5^, Volkan M. Çakir ^5^, Sonja Grunewald ^6^, Jan-Christoph Simon ^6^, Andreas Dietz ^7^, Dirk Dannenberger ^8^, Antje Garten ^9^, Johannes R. Lemke ^1^, Jürgen Schiller ^4^, Matthias Blüher ^10^, Pamela Arielle Nono Nankam ^11^, Ulrike Rolle-Kampczyk ^12^, Martin von Bergen ^12, 13^, Janet Kelso ^2^, Torsten Schöneberg ^5, *^

^1^ Institute of Human Genetics, University Medical Center Leipzig, Leipzig, 04103, Germany

^2^ Department of Evolutionary Genetics, Max Planck Institute for Evolutionary Anthropology, Leipzig, 04103, Germany

^3^ Institute of Biomedical Informatics, National Yang-Ming University, Taipei, 11221, Taiwan

^4^ Institute for Medical Physics and Biophysics, Medical Faculty, Leipzig University, 04107 Leipzig, Germany

^5^ Division of Molecular Biochemistry, Rudolf Schönheimer Institute of Biochemistry, Medical Faculty, Leipzig University, 04103 Leipzig, Germany

^6^ Department of Dermatology, Venereology and Allergology, University Medical Center Leipzig, 04103 Leipzig, Germany

^7^ Clinic for Otorhinolaryngology, Head and Neck Surgery, University Medical Center Leipzig, 04103 Leipzig, Germany

^8^ Leibniz Institute for Farm Animal Biology, Institute of Muscle Biology and Growth, Wilhelm-Stahl-Allee 2, 18196 Dummerstorf, Germany

^9^ Pediatric Research Center, University Hospital for Children and Adolescents, Leipzig University, 04103 Leipzig, Germany

^10^ Helmholtz Institute for Metabolic, Obesity and Vascular Research (HI-MAG) of the Helmholtz Zentrum München at the University of Leipzig and University Hospital Leipzig, Leipzig, Germany

^11^ Department of Endocrinology, Faculty of Medicine, University of Leipzig, 04103 Leipzig, Germany

^12^ Helmholtz Centre for Environmental Research GmbH, Department of Molecular Systems Biology, 04318 Leipzig, Germany

^13^ Institute of Biochemistry, Faculty of Life Sciences, Leipzig University, Bruederstr. 32, 04103 Leipzig, Germany

* Corresponding authors:

Diana Le Duc: [gabriela-diana.leduc@medizin.uni-leipzig.de](mailto:gabriela-diana.leduc@medizin.uni-leipzig.de); [diana_leduc@eva.mpg.de](mailto:diana_leduc@eva.mpg.de)

Torsten Schöneberg: Rudolf Schönheimer Institute of Biochemistry, Johannisallee 30, 04103 Leipzig, Germany; [schoberg@medizin.uni-leipzig.de](mailto:schoberg@medizin.uni-leipzig.de)

# Lipidomics

## Lipid extraction

Tissue samples were extracted using methyl-*tert*-butyl ether (MTBE) following the protocol of Matyash *et al*. ^1^ with slight modifications. Briefly, tissue samples were transferred to 1 ml methanol containing 1 mM butylhydroxytoluol (BHT) preventing oxidation in ball mill tubes (Precellys® ceramic-kit, CKMIX, Bertin GmbH, Frankfurt am Main, Germany) and homogenized using the tissue homogenizer Precellys® 24 (VWR, Radnor, Pennsylvania, USA) for 2 × 5 s at 5 500 rpm, then moved on immediately ice. The homogenized samples were transferred into glass vials, washed ball mill tubes twice with 1 ml MTBE and added 4 ml MTBE to the samples. Samples were incubated for 1 h at 4 °C and 800 rpm. To achieve phase separation, 1 ml aqua dest. was added to each sample followed by 10 minutes centrifugation at 4 °C and 3 500 rpm. To maximize the extraction yields, the pellets were subjected to a second extraction step with 2 ml MTBE and 0.5 ml aqua dest. and combined the obtained organic phases. MTBE was removed by evaporation under a gentle nitrogen steam. The samples were stored at -20 °C for further experiments.

## High-Performance Thin-Layer Chromatography (HPTLC) coupled to Electrospray ionization ion trap mass spectrometry (ESI-IT MS)

To overcome potential suppression effects, crude lipid extracts were separated by HPTLC and the individual lipid fractions (TAG, PC, and SM) were subsequently analyzed by means of electrospray ionization-ion trap mass spectrometry (ESI-IT MS).

HPTLC and ESI-IT MS measurements were performed according to Engel *et al*. ^2^ with slight modifications. Crude lipid extracts were dissolved in chloroform to yield 10 µg/µl solutions and 10 µl of each sample were sprayed onto an HPTLC silica gel 60 plate (Merck KGaA, Darmstadt, Germany) using a CAMAG^®^ Linomat 5 semi-automatic sample application system (CAMAG, Berlin, Germany). For the identification of TAGs, plates were developed in vertical TLC chambers with hexane/diethyl ether/acetic acid (80:20:1, v/v/v) as the solvent system ^3^. Separation of phospholipids (PLs) was performed by using chloroform/ethanol/water/triethylamine (30:35:7:35, v/v/v/v) as mobile phase. In all cases, lipids were visualized by dipping the entire plate in primuline (Direct Yellow 59, Sigma-Aldrich, Taufkirchen, Germany) dissolved in acetone/water (80:20, v/v, 50 mg/l). Upon illumination with UV light (366 nm), lipids were detected as colored spots. Lipid fractions were marked with a pencil, automatically eluted by a Plate Express™ TLC plate reader (Advion, Ithaca, NY, USA) with methanol as solvent and subsequently directly transferred into the ESI mass spectrometer.

ESI-IT MS was performed on an Amazon SL mass spectrometer (Bruker Daltonics GmbH, Bremen, Germany) by direct infusion using the following settings: spray voltage 4.5 kV, end plate offset 500 V, nebulizer gas 7.3 psi, drying gas (N_2_) 3 l/min, capillary temperature 180°C, flow rate 3 μl/min, sheath gas (He) flow rate 25 a.u. The spectra were recorded exclusively in the positive ion mode with enhanced resolution. For data acquisition and subsequent analysis, the Bruker "Trap Control" and "Data Analysis" version 4.1 software were used, respectively.

Representative ESI mass spectra of TAG fractions are depicted in Supplementary Fig. S1 and the assignment of the *m/z* values is shown in Supplementary Table S1.

**
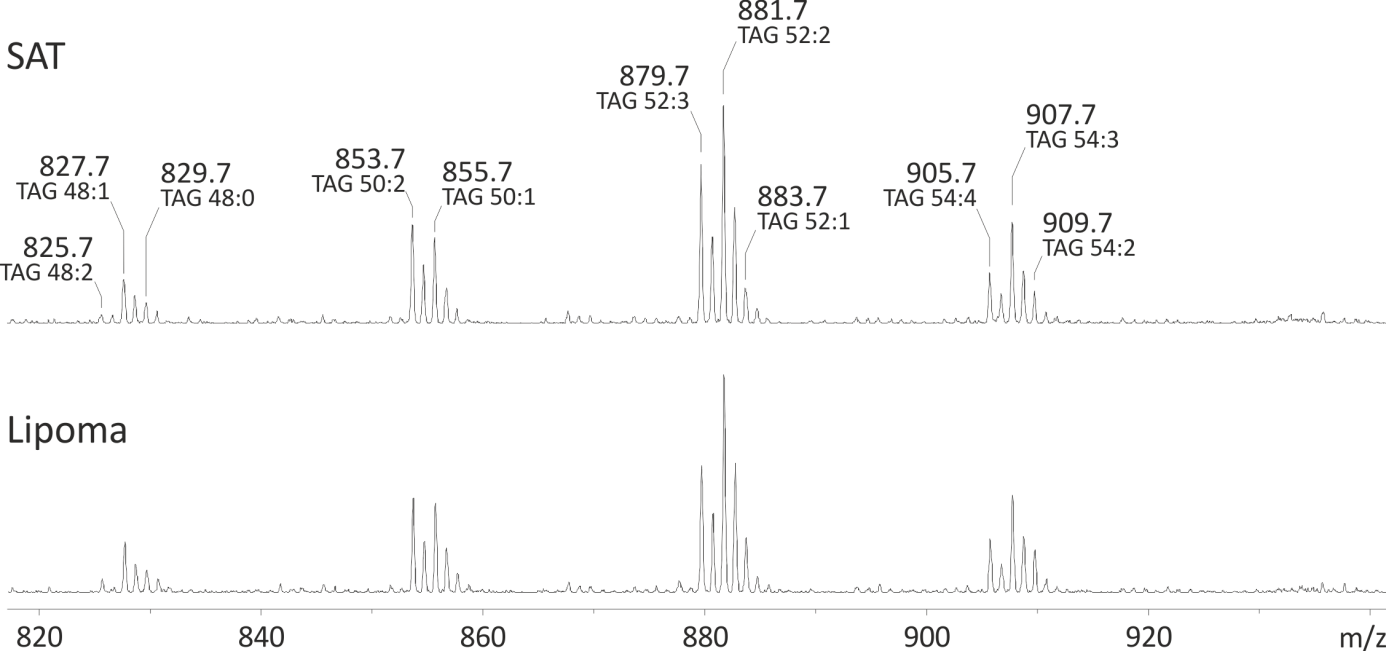
**

**Supplementary Fig. S1.** Representative ESI-IT mass spectra of isolated TAG fractions from organic extracts of selected example of subcutaneous adipose tissues (SAT) and corresponding lipoma tissue samples. Triacylglycerols (TAGs) species are exclusively detected as sodium adducts in both spectra. All peaks are marked by their *m/z* values and a detailed assignment of all observed peaks is available in Supplementary Table S1.

**Supplementary Table S1.** Assignment of the *m/z* values detected in the positive ion ESI-IT mass spectra of the SAT and corresponding lipoma samples.

| ***m/z ratio*** | ***Assignment*** |
| --- | --- |
| 825.7 | TAG 48:2+Na^+^ |
| 827.7 | TAG 48:1+Na^+^ |
| 827.7 | TAG 48:0+Na^+^ |
| 853.7 | TAG 50:2+Na^+^ |
| 855.7 | TAG 50:1+Na^+^ |
| 879.7 | TAG 52:3+Na^+^ |
| 881.7 | TAG 52:2+Na^+^ |
| 883.7 | TAG 52:1+Na^+^ |
| 905.7 | TAG 54:4+Na^+^ |
| 907.7 | TAG 54:3+Na^+^ |
| 909.8 | TAG 54:2+Na^+^ |

## Proton nuclear magnetic resonance (^1^H NMR)

^1^H NMR measurements were performed on a Bruker AVANCE-700 (Bruker, Rheinstetten, Germany) spectrometer operating at 700.13 MHz for ^1^H. All spectra were recorded at 310 K using a 5-mm inverse probe and the sample volume was 450 μl in all cases. Mixtures between deuterated chloroform and methanol were used to suppress the aggregation of lipids as far as possible. We used a 9.15 μs π/2 pulse for excitation. To allow for complete relaxation the delay between two scans was set to 10 s which is sufficient to compensate differences in T_1_ relaxation times (about 2–4 s). We verified this by measuring the T_1_ relaxation times in selected samples using the inversion recovery pulse sequence. We sampled 64 k points in the time domain. All spectra were corrected for baseline and phase distortions and calibrated using the residual proton resonance of methanol at 3.49 ppm. The lipid composition of the samples was determined by integrating the methyl (0.9 ppm), allylic (2.7 ppm), olefinic (5.3 ppm), and vicinal-olefinic (2.0 ppm) resonances.

Representative ^1^H NMR spectra are shown in Supplementary Fig. S2.


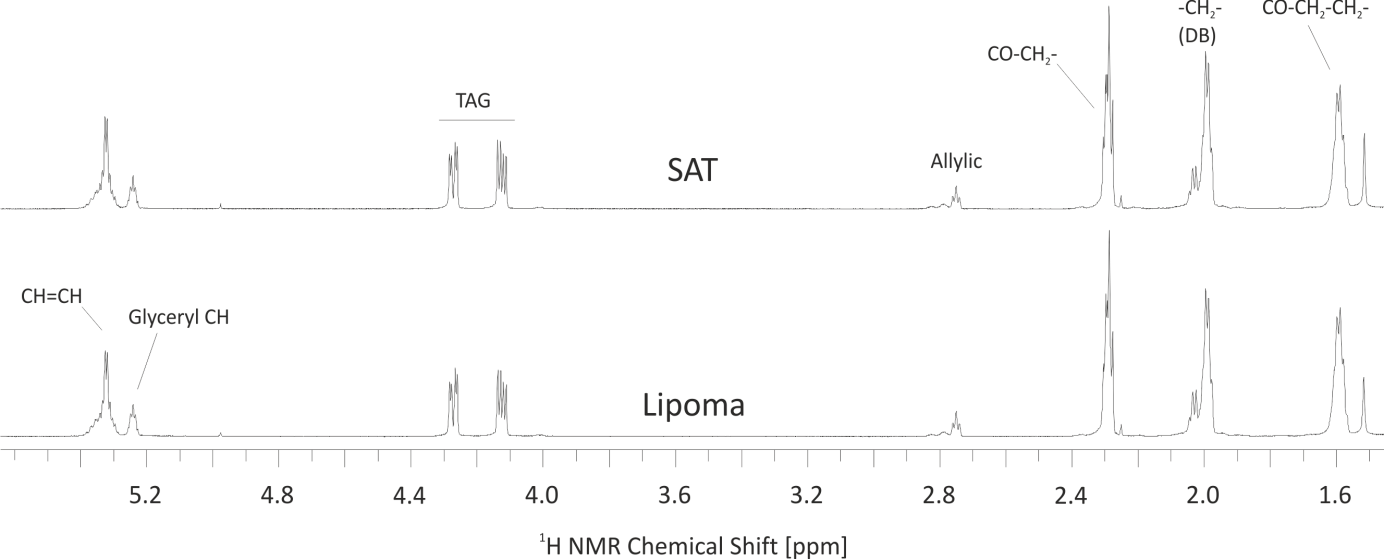


**Supplementary Fig. S2.** Crude organic extracts of SAT and lipoma were investigated via ^1^H NMR. Although NMR is by far less sensitive compared to MS and provides less accurate information about the lengths of the fatty acyl residues, it has the advantage that it is quantitative, i.e. the integral intensities are directly proportional to the concentration of the observed resonance. The NMR spectra are dominated by TAGs and the resonances of the most relevant functional groups can be easily assigned.

## Gas chromatography flame-ionization detection (GC-FID)

Independent GC-FID analysis were performed as previously described ^4^. All solvents contained 0.005 % (w/v) of BHT to prevent the oxidation of polyunsaturated fatty acids. The lipid extracts (vide supra) were re-dissolved in 300 μl of toluene and used an aliquot for methyl ester preparation. Next, 2 ml of 0.5 M sodium methoxide in methanol were added to the samples, which were shaken in a 60 °C water bath for 10 min. Subsequently, 1 ml of 14 % boron trifluoride in methanol was added to the mixture, which was then shaken for additional 10 minutes at 60 °C. Saturated NaHCO_3_ (2 ml) was added and the fatty acid methyl esters (FAMEs) were extracted three times in 2 ml of n-hexane. We reduced the solvent containing the FAMEs to dryness and resuspended the FAMEs in 100 μl of n-hexane, which was stored at −18 °C until analysis. The detailed fatty acid analysis was performed using capillary GC with a CP-Sil 88 CB column (100 m × 0.25 mm, Chrompack-Varian, Lake Forest, CA, USA) that was installed in a Perkin Elmer gas chromatograph Autosys XL with a flame ionization detector and split injection (Perkin Elmer Instruments, Shelton, USA). The initial oven temperature was 150 °C, which was held for 5 min; subsequently, the temperature was increased to 175 °C and then to 200 °C at a rate of 2 °C min^−1^ and held constant for 10 min. Finally, the temperature was increased to 225 °C at a rate of 1.5 °C min^−1^ and held constant for 25 min. Hydrogen was used as the carrier gas at a flow rate of 1 ml min^−1^. The standard FAME Mix from Sigma-Aldrich (Deisenhofen, Germany) and purchased individual methyl esters of 18:4n-3, 22:4n-6, and 22:5n-3 from Matreya (Pleasant Gap, USA) and methyl esters of 18:1trans-11 and 18:1cis-11 from Larodan Fine Chemicals (Malmö, Sweden) were used as references. The fatty acid profile was calculated using the internal standard method (19:0 as the internal standard). The method was calibrated using five different concentration levels ranging 18–376 ng/μl for each investigated fatty acid.

# Metabolomics

The composition of different metabolites was analyzed on *n*=4 lipoma and matched SAT samples using the Absolute*IDQ*® p150 Kit (Biocrates Life Science AG, Austria). The kit is validated according to the U.S. Food and Drug Administration (FDA) Guidance for Industry. It covers 188 metabolites from 6 compound classes (acyl carnitines, amino acids, glycerophospho- and sphingolipids, biogenic amines, and hexoses) that can be analysed using MS. However, due to used extraction method with MTBE, only 145 metabolites from 3 compound classes (acyl carnitines, glycerophospho- and sphingolipids) could be reliably determined. Preparation was carried out according to the manufacturer's instructions.

The liquid chromatography with tandem mass spectrometry (LC-MS/MS) analysis was run by multiple reaction monitoring (MRM) acquisition using a Waters Acquity Ultra-Performance Liquid Chromatography (UPLC) System (Waters, Eschborn, Germany) coupled with QTRAP 5500 (AB Sciex, Darmstadt, Germany). The analyses were performed by an isocratic Flow Injection Analysis Tandem Mass Spectrometry (FIA-MS-MS) method using an electrospray ionisation in positive and negative ionisation mode by 3 min runs. Mass spectra were analyzed with Analyst Software version 1.6.2 and validated by the MetVal software tool from the MetIDQ Software tool delivered by Biocrates Life Science AG. An automatic quality assessment was done using blanks, internal standards, and quality controls.

We observed minor changes mainly in the membrane lipid composition (Supplementary Fig. S3). To test whether the change in membrane lipid composition is indeed significant, we further analyzed phosphatidylcholines and sphingomyelins using HPTLC coupled with ESI MS (Supplementary Fig. S4) on all *n*=10 lipoma and matched SAT samples. As in the case of storage lipids (TAGs), only minor, not significant differences could be observed for PC and SM lipid classes.

**Supplementary Fig. S3.** Volcano plot of the measured metabolic compounds: in lipoma *vs*. the matched SAT (*n*=4). Some phosphatidylcholine (PC) show a marginally increased expression in lipoma.


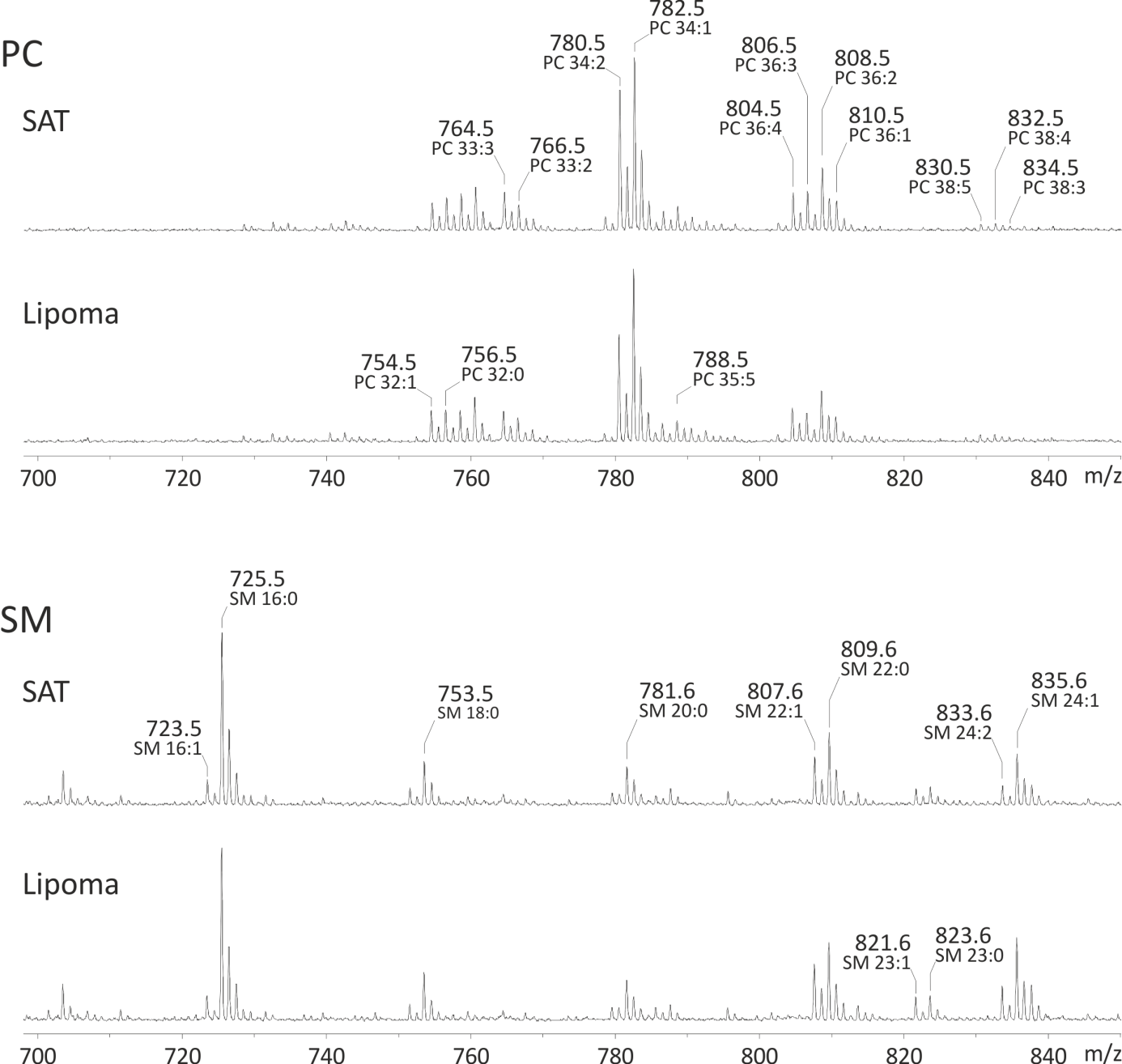


**Supplementary Fig. S4.** Representative ESI-IT mass spectra of phosphatidylcholine (PC) and sphingomyelin (SM) fractions of selected subcutaneous adipose tissue (SAT) and corresponding lipoma tissue samples recorded in positive ion mode. Lipid fractions were obtained by HPTLC. Only the different sodiated lipid species are labeled by their *m/z* values, while the proton adducts were not considered due to their lower abundance. Assignment of the corresponding *m/z* values is summed up in Supplementary Table S2.

**Supplementary Table S2.** Assignment of the PC and SM *m/z* values of the SAT and corresponding lipoma samples detected in the positive ion mode ESI-IT MS. Note that only sodium adducts (+Na^+^) are assigned.

| ***m/z ratio*** | ***Assignment*** | ***m/z ratio*** | ***Assignment*** |
| --- | --- | --- | --- |
| 754.5 | PC 32:1 | 723.5 | SM 16:1 |
| 756.5 | PC 32:0 | 725.5 | SM 16:0 |
| 764.5 | PC 33:3 | 753.5 | SM 18:0 |
| 766.5 | PC 33:2 | 781.5 | SM 20:0 |
| 768.6 | PC 33:1 | 807.5 | SM 22:1 |
| 780.5 | PC 34:2 | 809.6 | SM 22:0 |
| 782.5 | PC 34:1 | 821.6 | SM 23:1 |
| 786.6 | PC 35:6 | 823.6 | SM 23:0 |
| 788.6 | PC 35:5 | 833.6 | SM 24:2 |
| 790.6 | PC 35:4 | 835.6 | SM 24:1 |
| 792.5 | PC 35:3 | 837.6 | SM 24:0 |
| 804.5 | PC 36:4 |  |  |
| 806.5 | PC 36:3 |  |  |
| 808.5 | PC 36:2 |  |  |
| 810.6 | PC 36:1 |  |  |
| 830.5 | PC 38:5 |  |  |
| 832.6 | PC 38:4 |  |  |
| 834.6 | PC 38:3 |  |  |

# Transcriptomics

## RNA extraction and sequencing

For library preparation and sequencing, total RNA was extracted from *n*=15 lipomas and the matched normal SAT using RNeasy® Lipid Tissue Kit (Qiagen). The quality and quantity of the purified RNA was determined by measuring the absorbance at 260/280 nm and 260/230 nm using a NanoDrop spectrophotometer and by gel electrophoresis to visualize the 28S and 18S rRNA bands as indicators of total RNA integrity. The ratio of 28S/18S intensity was at least 2 to include the sample for library preparation. Indexed cDNA libraries were constructed with an average insert size of 300 bp using the TruSeq RNA sample preparation kits v2 (Illumina, San Diego, CA). Libraries were sequenced on an Illumina HiSeq platform as 101 bp paired-end reads to an average of 46.9 million reads per library. On average, more than 95.2 % of the reads had a quality score over 30 (Q30 Phred-scale).

## Processing of RNA reads

RNA-seq reads were demultiplexed, trimmed of adaptors, and mapped with STAR (version 2.6.1d) ^5^ to the GRCh38 genome assembly. To detect gene fusions and increase the sensitivity for novel splice junctions the following options in addition to default parameters were used: --chimSegmentMin 10 --chimOutType WithinBAM SoftClip --chimJunctionOverhangMin 10 --chimScoreMin 1 --chimScoreDropMax 30 –chimScoreJunctionNonGTAG 0 --chimScoreSeparation 1 --alignSJstitchMismatchNmax 5 -1 5 5 --chimSegmentReadGapMax 3.

### Gene fusion analysis

Arriba (https://github.com/suhrig/arriba/) was used to detect gene fusions from RNA-Seq data. Briefly, the fusion candidates are generated by STAR ^5^ and collected in the chimeric alignment files. To reduce the number of artefacts Arriba removes a list of known fusion events which includes recurrent alignment artifacts and transcripts which are present in healthy tissue, such as read-through fusions between neighboring genes, circular RNAs, and other non-canonically spliced transcripts. Using standard parameters homopolymers, hairpins, inconsistently clipped reads, mismatches or reads with long gaps are removed, since they are considered artefacts. To each prediction Arriba assigns a confidence of low, medium, or high. The confidence reflects the likelihood that: the transcript is aberrant (not present in healthy tissue), there is a genomic rearrangement, and there is no artifact. Arriba was run sequentially on all samples and only fusion events with high confidence level were considered.

### Differential gene expression analysis

The transcription level of each gene was determined using htseq-count ^6^ followed by differential analysis on gene count data with DESeq2 ^7^. This R package implements shrinkage estimation for dispersions and fold changes, which improves the stability and reproducibility of results compared to maximum-likelihood-based solutions. The initial assumption is that genes of similar average expression level have similar dispersion. A curve is fitted in the plot of dispersion values over the average expression level of the genes. Using an empirical Bayes approach the dispersion values are shrunk to obtain estimates. The shrinkage is dependent on how close true dispersion values tend to be to the fit and on the degrees of freedom: as the sample size increases, the shrinkage decreases and eventually becomes negligible. Thus, the method is applicable for analyses of differential expression (DE) with few replicates as well as for large sample-sized studies.

Gene expression analysis was run on the two groups, lipoma and SAT, accounting for the matched samples. The initial analysis was performed using all *n*=15 samples to test whether the inclusion of potential multiple genomic backgrounds, and consecutive pathomechanisms, may lead to additional noise. To narrow down the genomic background variation and delineate pathways involved in fat accumulation, the analysis was further focused on two lipoma groups: with (*n*=4) and without *HMGA2* fusion genes (*n*=4). The other 7 remaining samples displayed high- or medium-confidence fusions involving different gene partners. To avoid noise, these samples were not included in further analyses. Analyses were focused on the shared up and down-regulated genes between lipomas without fusions and with *HMGA2*-fusions and corrected for multiple testing using Benjamini-Hochberg method.

Differentially expressed GPCRs based on the analysis including all samples are presented in suppl. Table, GPCRs coupling. To decide whether GPCRs are expressed in adipose tissue or immune cells we inquired their expression levels based on GTEx (<https://www.gtexportal.org/home/>). Genes with TPM (transcripts per million) > 1.5 in subcutaneous adipose tissue were considered to be expressed in adipocytes. Thus, there were 9 Gi-coupled GPCRs, all up regulated in lipoma and one Gs-coupled GPCR, also up-regulated. This lends further support to a reduction in the intracellular cAMP.

### Protein-protein interaction networks and gene ontology analysis

To better understand the pathomechanism of lipoma, conventional functional enrichment analysis for DE genes was performed. The functional annotations of genes were obtained from Gene Ontology (GO) ^8, 9^. The functions of studied DE genes (*p*-value < 0.05) were identified as the significantly enriched functions in which the DE genes are involved. The significance is established by *p*-value from the hypergeometric test. The hypergeometric distribution is described as below:

$P\left( X=k \right)=\frac{(\begin{matrix} m \\ k \end{matrix})(\begin{matrix} N-m \\ n-k \end{matrix})}{(\begin{matrix} N \\ n \end{matrix})}$ (1)

where *X* denotes the evaluated function; *N* represents the number of GO annotated genes; *m* indicates the number of DE genes; *n* represents the number of genes with the evaluated function; *k* indicates the number of DE genes with the evaluated function. To enhance the functional relationship between DE genes involved in the identified functions, the protein interaction and a network-wise functional enrichment analysis were incorporated to discover the functional modules within DE genes ^10^. The source of the human protein-protein interaction (PPI) data is InBio Map ^11^. The network-wise functional enrichment analysis is modified from the conventional way. The significance of the tested function is based on *p*-values produced from a modified hypergeometric test. The modified hypergeometric distribution is described as below:

$P_{e}\left( X=k_{e} \right)=\frac{(\begin{matrix} m_{e} \\ k_{e} \end{matrix})(\begin{matrix} N_{e}-m_{e} \\ n_{e}-k_{e} \end{matrix})}{(\begin{matrix} N_{e} \\ n_{e} \end{matrix})}$ (2)

e is the abbreviation of the PPI. Each symbol has the same meaning as in the conventional hypergeometric distribution, but the counting objects are changed from genes to functional PPIs. The functional PPIs are PPIs formed by the two genes involved in the same functions. This approach revealed the significant protein interaction functional modules in which the DE genes were involved. These two *p*-values were adjusted by the Benjamini and Hochberg multiple testing procedures to control the false discovery rate (FDR) ^12^.

We aimed to inquire shared functional modules between lipomas without fusions and with *HMGA2*-fusions. To increase the power of functional module identification, the protein interaction partners of the common DE genes were incorporated. Thus, the functional modules were formed by the common DE genes and their protein interaction partners. Notably, the identified functional modules must contain significantly enriched DE genes to retain the influence of DE genes. Yet, the expanded functional modules formed by DE genes and their interaction partners can incorporate interactions not formed by DE genes. This analysis is only indicative for shared functions, since we cannot establish significance based on the *p*-values derived from the enrichment of functional interactions.

To further inquire what pathways are significantly dysregulated, an enrichment analysis of gene ontology biological process categories was performed ^13^. We considered results to be significant for an FDR < 0.05.

We finally inquired the KEGG ^14^ pathway regulation of lipolysis in adipocytes (map 04923) and assessed the genes that showed DE in lipomas without fusions and the *HMGA2*-fusion-group. The expression profile of the gene classes was considered to follow a binomial distribution and calculated the probability of observing differentially expressed genes as $\binom{n}{k}p^{k}{(1-p)}^{n-k}$, where *n*=number of gene classes in the pathway, *k*=number of classes showing differential gene expression, and *p*=0.5, the probability of a gene class to show DE.

### Splice site analysis

We inquired whether differential splicing is involved in lipoma etiology. To this end we compared each lipoma to the matched SAT.

LeafCutter ^15^ was used to detect aberrant splicing events. Because we were facing different genomic background in each case we adjusted LeafCutter to this setting by testing sequentially for each patient, the lipoma against the matched normal SAT (min_samples_per_group=1; min_samples_per_intron=1). Further, we also modified the parameters to detect rare clusters and capture junctions that are unique to lipoma (minclureads=30; maxintronlen=500 000; mincluratio=1e-5).

The only gene that showed differential splicing that withstood multiple testing correction and was shared by multiple samples (9 of 15) was *UBC*. *UBC* is composed of two exons, such that the majority of the alternative splicing events were not in the canonical site, but termed cryptic (Suppl. Figs. S5 a–d). However, on closer inspection of the gene and the resulting peptide, we noted that the peptide is formed by 9 subunits, each consisting of 76 amino acids. Since the read length is only 101 bp, we cannot reliably detect the splicing position. While some of the reads point to a shorter peptide subunit, the result would have to be validated using longer read lengths. Yet, Sanger technology cannot be employed, since primers would bind in multiple locations within the gene. Since we cannot verify the finding, we are only reporting this result in this Supplementary Material and do not further refer to this in the main text.

**Supplementary Fig. S5.** Representative splicing variants of the UBC gene in 4 (a–d) lipoma samples. The majority of the splicing events are in cryptic sites (pink color), as opposed to the canonical site (red color). LIPO = lipoma, SAT = subcutaneous adipose tissue.

### Transcription factors analysis

EPEE ^16^ was used to perform differential analysis of transcription factor (TF) activity from gene expression data. Briefly, on the data mapped with STAR (version 2.6.1d) ^5^ to the GRCh38 genome assembly we ran StringTie ^17^ to calculate gene abundances as TPMs (transcripts per million). We then ran EPEE on all samples using as reference human network the SAT TFs from FANTOM5 ^18^. We then repeated the analysis using only samples from lipomas without gene fusions and with *HMGA2*-fusions, respectively. Finally, we averaged the scores from all three runs to rank the TFs that may have highest influence on the expression profiles. Since the reached scores were not very high (Suppl. Fig. S6), we conclude that the influence of these TFs on gene expression is rather limited.

**Supplementary Fig. S6.** Transcription factors with highest ranking according to the mean score from three analyses: including all lipoma samples, samples with *HMGA2*-fusions and samples without fusions. Positive scores denote activity in lipomas, while negative scores show higher activity in matched SAT.

# Supplementary References

1. Matyash V, Liebisch G, Kurzchalia TV, Shevchenko A, Schwudke D. Lipid extraction by methyl-tert-butyl ether for high-throughput lipidomics. *Journal of lipid research* 2008; **49**(5)**:** 1137-46.

2. Engel KM, Sampels S, Dzyuba B, Podhorec P, Policar T, Dannenberger D *et al.* Swimming at different temperatures: The lipid composition of sperm from three freshwater fish species determined by mass spectrometry and nuclear magnetic resonance spectroscopy. *Chemistry and physics of lipids* 2019; **221:** 65-72.

3. Zhang S, Wang Y, Cui L, Deng Y, Xu S, Yu J *et al.* Morphologically and Functionally Distinct Lipid Droplet Subpopulations. *Scientific reports* 2016; **6:** 29539.

4. Dannenberger D, Nuernberg G, Nuernberg K, Will K, Schauer N, Schmicke M. Effects of diets supplemented with n–3 or n–6 PUFA on pig muscle lipid metabolites measured by non-targeted LC–MS lipidomic profiling. *Journal of Food Composition and Analysis* 2017; **56:** 47-54.

5. Dobin A, Davis CA, Schlesinger F, Drenkow J, Zaleski C, Jha S *et al.* STAR: ultrafast universal RNA-seq aligner. *Bioinformatics (Oxford, England)* 2013; **29**(1)**:** 15-21.

6. Anders S, Pyl PT, Huber W. HTSeq--a Python framework to work with high-throughput sequencing data. *Bioinformatics (Oxford, England)* 2015; **31**(2)**:** 166-9.

7. Love MI, Huber W, Anders S. Moderated estimation of fold change and dispersion for RNA-seq data with DESeq2. *Genome biology* 2014; **15**(12)**:** 550.

8. Consortium TGO. The Gene Ontology Resource: 20 years and still GOing strong. *Nucleic acids research* 2019; **47**(D1)**:** D330-d338.

9. Ashburner M, Ball CA, Blake JA, Botstein D, Butler H, Cherry JM *et al.* Gene ontology: tool for the unification of biology. The Gene Ontology Consortium. *Nature genetics* 2000; **25**(1)**:** 25-9.

10. Lin CC, Hsiang JT, Wu CY, Oyang YJ, Juan HF, Huang HC. Dynamic functional modules in co-expressed protein interaction networks of dilated cardiomyopathy. *BMC systems biology* 2010; **4:** 138.

11. Li T, Wernersson R, Hansen RB, Horn H, Mercer J, Slodkowicz G *et al.* A scored human protein-protein interaction network to catalyze genomic interpretation. *Nature methods* 2017; **14**(1)**:** 61-64.

12. Benjamini Y, Hochberg Y. Controlling the false discovery rate: a practical and powerful approach to multiple testing. *Journal of the Royal statistical society: series B (Methodological)* 1995; **57**(1)**:** 289-300.

13. Szklarczyk D, Morris JH, Cook H, Kuhn M, Wyder S, Simonovic M *et al.* The STRING database in 2017: quality-controlled protein-protein association networks, made broadly accessible. *Nucleic acids research* 2017; **45**(D1)**:** D362-d368.

14. Kanehisa M, Goto S. KEGG: kyoto encyclopedia of genes and genomes. *Nucleic acids research* 2000; **28**(1)**:** 27-30.

15. Li YI, Knowles DA, Humphrey J, Barbeira AN, Dickinson SP, Im HK *et al.* Annotation-free quantification of RNA splicing using LeafCutter. *Nature genetics* 2018; **50**(1)**:** 151-158.

16. Amin V, Agac D, Barnes SD, Cobanoglu MC. Accurate differential analysis of transcription factor activity from gene expression. *Bioinformatics (Oxford, England)* 2019.

17. Pertea M, Pertea GM, Antonescu CM, Chang TC, Mendell JT, Salzberg SL. StringTie enables improved reconstruction of a transcriptome from RNA-seq reads. *Nature biotechnology* 2015; **33**(3)**:** 290-5.

18. Forrest AR, Kawaji H, Rehli M, Baillie JK, de Hoon MJ, Haberle V *et al.* A promoter-level mammalian expression atlas. *Nature* 2014; **507**(7493)**:** 462-70.
